# Developing a Telephone Assessment of Physical Activity (TAPA) Questionnaire for Older Adults

**Published:** 2007-12-15

**Authors:** Charles J Mayer, Lesley Steinman, Barbara Williams, James LoGerfo, Tari D Topolski

**Affiliations:** University of Washington Health Promotion Research Center, University of Washington Department of Family Medicine; University of Washington Health Promotion Research Center, Seattle, Washington; University of Washington Health Promotion Research Center, Seattle, Washington; University of Washington Health Promotion Research Center, Seattle, Washington; Seattle Quality of Life Group, University of Washington, Seattle, Washington

## Abstract

**Introduction:**

We report on development and preliminary validation of a brief, telephone-based measurement tool for assessing physical activity in older adults. The Telephone Assessment of Physical Activity (TAPA) questionnaire is based on the University of Washington Health Promotion Research Center's Rapid Assessment of Physical Activity (RAPA), a written questionnaire.

**Methods:**

The Rapid Assessment of Physical Activity questionnaire was modified to permit interviewers to administer it as a telephone interview. We retained its scoring levels and interpretation. The pilot test of the telephone version assessed the questionnaire's ease of administration and construct validity in a community-based sample of older adults. Spearman rho and kappa statistics were computed for comparison with the Rapid Assessment of Physical Activity questionnaire and the Community Healthy Activities Model Program for Seniors questionnaire.

**Results:**

Thirty-four older adults completed the telephone assessment. A Spearman rho of 0.74 and a kappa statistic of 0.48 were found between TAPA and the written RAPA.

**Conclusion:**

The pilot test demonstrated that the TAPA questionnaire is a promising instrument for use as a brief, telephone-based questionnaire for assessing physical activity in older adults.

## Introduction

Physical activity has been shown to assist older adults in managing chronic conditions and to delay decline in their physical and mental health (1). Currently, however, reports show that fewer than 20% of U.S. adults aged 64 or older engage in the U.S. Surgeon General's recommended levels of physical activity (2), and only 11% engage in strength training (3).

The Rapid Assessment of Physical Activity (RAPA) questionnaire was designed to provide clinicians with a tool for quickly assessing the level of physical activity of their older adult patients (4). It was developed following an extensive review and evaluation of existing written questionnaires, which were found either to be too long or to lack sufficient sensitivity for measuring physical activity in older adults. RAPA was found to be reliable and valid compared with the longer, validated Community Healthy Activities Model Program for Seniors (CHAMPS) questionnaire (4,5). However, one drawback to RAPA's use outside the clinical setting is its highly visual format, which is not amenable to a telephone-based assessment of physical activity. This study was designed to address this limitation of RAPA by adapting it for use in telephone-based surveys of physical activity.

Other telephone-based physical activity surveys have been used and validated for general use; however, these surveys were not designed to address specific aspects of physical activity among older adults, for example, capturing lighter activities, such as walking leisurely, light vacuuming, light yard work, or light exercise such as stretching (5-7). Telephone-based surveys could be an ideal means of assessing physical activity in older adults, given the many challenges that prevent researchers from evaluating and monitoring this population group, such as the dependence of seniors on others for transportation to a research site. Disabilities often preclude travel to appointments with health care providers and to research sites. Furthermore, evaluating physical activity during visits to health care providers is often difficult because of the large number of competing health issues to be addressed.

We will discuss the process by which we adapted and developed a new telephone-based physical activity survey for older adults and our preliminary findings from a pilot test of the survey. We compare the Telephone Assessment of Physical Activity (TAPA) with RAPA, the system on which it was modeled, and to CHAMPS for criterion validity. Because scoring for both TAPA and RAPA are the same, we hypothesized that if both compare equally well with the CHAMPS instrument, an argument for using TAPA and RAPA interchangeably could be made. Our goal is to help researchers, clinicians, and public health practitioners quickly assess and monitor levels of physical activity in older adults.

## Methods

### Study design, sample, and setting 

In our study's cross-sectional design, we recruited older adults from the greater Seattle area using advertisements at senior centers, congregate meal sites, and senior public housing. We distributed flyers and used senior services representatives to recruit participants. Criteria for inclusion were being aged 50 years or older, English-speaking, and having the ability to answer questions regarding physical activity on both a written questionnaire mailed to participants and in a telephone survey. Assistance in filling out the written survey was offered to anyone who needed help because of physical disability (e.g., poor vision, arthritic pain in the hands). We excluded from the study those who were unable to answer questions because of significant cognitive impairment (e.g., Alzheimer's disease) or severe acute illnesses (e.g., active heart failure).

The CDC-funded Health Promotion Research Center (HPRC) at the University of Washington in Seattle oversaw development and adherence to the study protocol. A research assistant with a master's degree in public health mailed and received all the written questionnaires and administered all the telephone physical activity questionnaires. An effort was made to include underrepresented participants, including men, people of color, and less active seniors.

### Questionnaire development 

HPRC researchers, along with members of the RAPA development team, began by adapting the RAPA questions to a telephone survey format. Participants who met the eligibility criteria and gave oral consent during a screening telephone call were then administered the TAPA. After finishing the survey, the research assistant gained qualitative tool performance information by asking the following questions: We are developing this survey to use with health care and social service providers who work with older adults. Do you have any comments about the survey (probe about satisfaction, ease of use, acceptability, comprehension)? What did you like about the survey? What could be improved? One week after TAPA administration, the written versions of RAPA and CHAMPS were either mailed to the participant's home or arrangements were made to meet the participant in person to administer the questionnaires orally. Information gathered by the research assistant was used in an iterative process to allow successive improvements to the questionnaire.

We tested two earlier versions of TAPA to improve its ease of use and understandability. The earlier versions had more complicated sentence structure. We found that participants understood and more easily responded to questions with fewer concepts to consider, leading us to subdivide some of our questions. For example, in version 2, question 4 reads "I do moderate physical activities every week, but less than 30 minutes per day, 5 days per week. Does this describe you?" In the final version, we separated this question into two questions, 4a and 4b (see Appendix); "I do some moderate physical activities every week, but less than 30 minutes per day. Does this describe you?" and "I do some moderate physical activities every week, but less than 5 days per week. Does this describe you?" A total of two pilot versions were administered during this iterative process. The questionnaire was administered between August 2005 and March 2007. The University of Washington Human Subjects Division approved all procedures, and participants received a nominal gratuity of $15 to thank them for participating.

Scoring of RAPA and TAPA was based on physical activity criteria derived from the Surgeon General's recommendations (2). One point is given for "sedentary level of activity," two for "underactive," three for "active but does not meet standard recommendations," four for "meets standard recommendations." CHAMPS scoring is based on caloric energy expended in moderate-intensity physical activities having a metabolic equivalent value of ≥3.0 (4).

### Analysis 

To assess how well TAPA captured the physical activity level of older adults, we compared it with the two written questionnaires, RAPA and CHAMPS. In initial analyses we looked at the agreement in levels of physical activity (sedentary to active), from TAPA and RAPA. We then analyzed the participants' answers to TAPA and RAPA for their relationship to CHAMPS, both in calories scored as a continuous variable and in meeting or exceeding the Surgeon General's physical activity recommendations. CHAMPS activities were scored as a continuous variable by determining moderate physical activity calories per week. Participants met the physical activity recommendation if they reported in CHAMPS that they engaged in moderate physical activities at least 5 days per week for a total of 3 or more hours per week or engaged in vigorous physical activities at least 3 days per week for a total of 1 or more hours per week. We assessed criterion validity by calculating a Spearman rho. Scoring instructions are described in the Appendix. Stata 9 software (StataCorp LP, College Station, Texas) was used for this analysis.

## Results

Thirty-six participants completed the TAPA telephone survey. Of those who completed TAPA, 34 also completed RAPA and CHAMPS. Participants were aged 63 to 92 years (mean age 75), were mostly female (62%), and represented a diverse sample of minority groups (Table 1). TAPA and RAPA each took 5 to 10 minutes to administer compared with 30 to 40 minutes for CHAMPS.

Table 2 shows the percentage of participants for each level of activity and compares responses from the TAPA and RAPA questionnaires. For both questionnaires, the four activity levels were fairly well distributed with a slightly greater percentage of participants meeting the Surgeon General's physical activities criteria for being sedentary or underactive (2).

The Spearman rho showed a moderately strong correlation of 0.738 (*P =* .001) between TAPA and RAPA (Table 3). A kappa statistic of 0.463 (*P =* .001) showed moderate agreement above chance between the same two questionnaires. TAPA, with a Spearman rho of 0.672 (*P =* .001) and a kappa statistic of 0.526 (*P =* .001), did not perform as well as CHAMPS. RAPA also did not perform as well as CHAMPS, with a Spearman rho of 0.663 (*P =* .001) and a kappa statistic of 0.398 (*P =* .001).

## Discussion

Our study begins to address the existing need among researchers, clinicians, and public health practitioners for a telephone-based physical activity assessment tool for older adults that is brief and effective. TAPA was developed using the strengths of the written RAPA questionnaire and going through two piloted versions in order to improve instrument quality. We designed the TAPA survey to err on the side of participants not meeting physical activity criteria when they actually met criteria; that is, to overestimate the false negative. Like RAPA, TAPA was designed to assess light activity that does not meet the CDC guidelines of 30 minutes or more of moderate physical activity on every day or on most days of the week (2).

TAPA is an easy-to-administer instrument that has demonstrated acceptability to a wide range of older adults. Though TAPA was not validated by a physical measurement, our study shows good agreement with RAPA. The TAPA and CHAMPS Spearman rho and kappa statistic were consistent with the RAPA and CHAMPS findings. This suggests that TAPA and RAPA may be equally effective in assessing physical activity of older adults in clinical practice.

There are several limitations to this study. The order of question iteration was not changed during the course of our study. This design flaw did not allow us to determine whether the order of the questions affected the strength of the comparisons. TAPA was not validated using an observable measure of physical activity. A sample size of 34, though diverse in both ethnicity and activity level, was not reflective of the Seattle population as a whole and may not be large enough to make any conclusive statements about TAPA. TAPA's generalizability may also be limited because our sample of seniors engaged in relatively high levels of physical activity compared with seniors in other published reports, which estimate that over 40% of the older U.S. adult population is completely sedentary (8). In addition, TAPA's effectiveness as a monitoring tool was not ascertained. This tool was used only in a cross-sectional analysis, and further research will be required to determine whether it is a competent resource for measuring change over time.

## Conclusion

TAPA is a brief, easy-to-administer, telephone-based survey developed in a diverse community setting. It has the same scoring and interpretive characteristics as RAPA; however, neither has been tested against a gold-standard physical measurement.

TAPA represents a good start at developing a physical activity assessment tool for older adults that is brief, easy to administer, and telephone-based. Such a tool will play an increasingly important role as the geriatric population increases and greater clinical and public health emphasis is placed on physical activity and on physical activity research.

TAPA needs further validation, including validation in a larger sample that includes a more sedentary group, and assessment of its ability to detect change over time. The next steps in development of TAPA include a larger study with similar outcome measures and a validation study with a physical measurement instrument (e.g., pedometer, accelerometer, gas exchange measurement device).

## Figures and Tables

**Table 1:** Demographic Characteristics of Study Participants, TAPA, RAPA, and CHAMPS (N = 34), August 2005 – March 2007

Characteristics	Value
Age, mean y (range)	75 (63-92)
Female sex	62%
BMI, mean (range)	24 (19-33)
**Race/ethnicity**	
White	12%
Asian/Native Hawaiian or other Pacific Islander	35%
Black/African American	26%
Hispanic or Latino	14%
American Indian/Alaskan Native	0%
** **Other or unknown	12%

TAPA indicates Telephone Assessment of Physical Activity questionnaire; RAPA, Rapid Assessment of Physical Activity questionnaire; CHAMPS, Community Healthy Activities Model Program for Seniors; BMI, body mass index.

**Table 2:** Percentage of Participants at Each Physical Activity Level, TAPA and RAPA,  August 2005 – March 2007 (N = 34)

Activity Level	TAPA (%)	RAPA (%)
Sedentary	26	26
Underactive	35	32
Active, does not meet standard	21	18
Active, meets standard	18	24

TAPA indicates Telephone Assessment of Physical Activity questionnaire; RAPA, Rapid Assessment of Physical Activity questionnaire.

**Table 3:** Comparison of TAPA, RAPA, and CHAMPS for All Physical Activity Levels and for Meeting U.S. Surgeon General's Physical Activity Recommendations, August 2005 – March 2007

Comparison (N = 34)	Spearman rho for Physical Activity^a^	Kappa Statistic for Relationship to Surgeon General's Physical Activity Recommendations b
TAPA vs RAPA	0.738 (*P* = .001)	0.463 (*P *= .001)
TAPA vs CHAMPS	0.672 (*P* = .001)	0.526 (*P* = .001)
RAPA vs CHAMPS	0.663(*P* = .001)	0.398 (*P* = .001)

TAPA indicates Telephone Assessment of Physical Activity questionnaire; RAPA, Rapid Assessment of Physical Activity questionnaire; CHAMPS, Community Healthy Activities Model Program for Seniors.

a Comparisons between TAPA and RAPA in this column have a range of 1–4 (1 indicates sedentary; 2, underactive; 3, active but does not meet standard; and 4,active and meets standard). Comparisons between TAPA and CHAMPS and RAPA and CHAMPS are based on calories expended per week of moderate activities (range, 0–7809).

b Comparisons for meeting Surgeon General's physical active recommendations (7) based on questionnaire responses.

## Appendix: Telephone Assessment of Physical Activity (TAPA) Questionnaire

### TAPA 1: Aerobic

I am going to ask you about the amount and level of physical activity you usually do. In this survey, we define physical activities as activities where you move and increase your breathing or heart rate. These are activities you do for pleasure, work, or for getting around.

I will read a statement about activities, and you can tell me whether the statement describes you by answering *yes* or *no*. For example,

SAMPLE	I am over 50 years old. Does this describe you?	**Yes □**	**No □**	**Not Sure □**

Do the best you can to answer using the yes/no format; at the end of the survey we can talk about specific activities.

The first statement is

1	I rarely or never do any physical activities. Does this describe you?	**Yes □**	**No □**	**Not Sure □**

The next statements are about three types of activities: light, moderate, and vigorous. Light activities are activities when your heart beats only slightly faster than normal and you can still talk and sing during them. Some examples of light activities are walking leisurely, light vacuuming, light yard work, or light exercise such as stretching. Here are two statements about light activity.

2a	I do some **light** physical activities, but not every week. Does this describe you?	**Yes □**	**No □**	**Not Sure □**
3	I do some **light** physical activity every week. Does this describe you?	**Yes □**	**No □**	**Not Sure □**

Next are moderate activities. Moderate activities are activities when your heart beats faster than normal. You can still talk but not sing during such activities. Some examples of moderate activities are fast walking, aerobics class, strength training, or swimming gently. I have four statements about moderate activities. The first one is

2b	I do some **moderate** physical activities, but not every week. Does this describe you?	**Yes □**	**No □**	**Not Sure □**
4a	I do some **moderate** physical activities every week, **BUT** less than 30 minutes per day. Does this describe you?	**Yes □**	**No □**	**Not Sure □**
4b	I do some **moderate** physical activities every week, **BUT** less than 5 days per week. Does this describe you?	**Yes □**	**No □**	**Not Sure □**
6	I do 30 minutes or more per day of **moderate **physical activities, 5 or more days per week. Does this describe you?	**Yes □**	**No □**	**Not Sure □**

The next three statements are about vigorous activities. Vigorous activities are activities when your heart rate increases a lot. You typically can't talk or your talking is broken up by large breaths. Some examples of vigorous activities are jogging, running, using a stair machine, or playing tennis, racquetball, badminton, or pickleball. The first statement is

5a	I do some **vigorou**s physical activities every week, **BUT** less than 20 minutes per day. Does this describe you?	**Yes □**	**No □**	**Not Sure □**
5b	I do some **vigorou**s physical activities every week, **BUT** less than 3 days per week. Does this describe you?	**Yes □**	**No □**	**Not Sure □**
7	I do 20 minutes or more per day of **vigorous**physical activities, 3 or more days per week. Does this describe you?	**Yes □**	**No □**	**Not Sure □**

### TAPA 2: Strength & Flexibility

And finally, I have two statements about strengthening and stretching activities. First,

1	I do activities to increase muscle **strength**, such as lifting weights or calisthenics, once a week or more. Does this describe you?	**Yes □**	**No □**	**Not Sure □**
2	I do activities to improve **flexibility**, such as stretching or yoga, once a week or more. Does this describe you?	**Yes □**	**No □**	**Not Sure □**

Are there activities that you do that reflect physical activity that we may have not captured in this survey?

(Write in response)

This concludes my questions. Thank you.

### TAPA 1: Aerobic, Scoring Instructions

To score, choose the question with the highest score with an affirmative response. Any number less than 6 is suboptimal.

For scoring or summarizing categorically:

Score as sedentary:

I rarely or never do any physical activities.

Score as underactive:

I do some light physical activities, but not every week, *or* I do some moderate physical activities, but not every week.

I do some light physical activity every week.

Score as underactive regular:

I do moderate physical activities every week, but less than 5 days per week or less than 30 minutes at a time.

I do vigorous physical activities every week, but less than 3 days per week or less than 20 minutes at a time.

Score as active:

I do 30 minutes or more per day of moderate physical activities, 5 or more days per week.

I do 20 minutes or more per day of vigorous physical activities, 3 or more days per week.

### TAPA 2: Strength & Flexibility, Scoring Instructions

*(Note: The authors made no analysis of TAPA 2 but present the scoring instructions in parentheses to make the complete TAPA questionnaire available to readers.)*

I do activities to increase muscle strength, such as lifting weights or calisthenics, once a week or more. (1)

I do activities to improve flexibility, such as stretching or yoga, once a week or more. (2)

Both. (3)

None (0)
